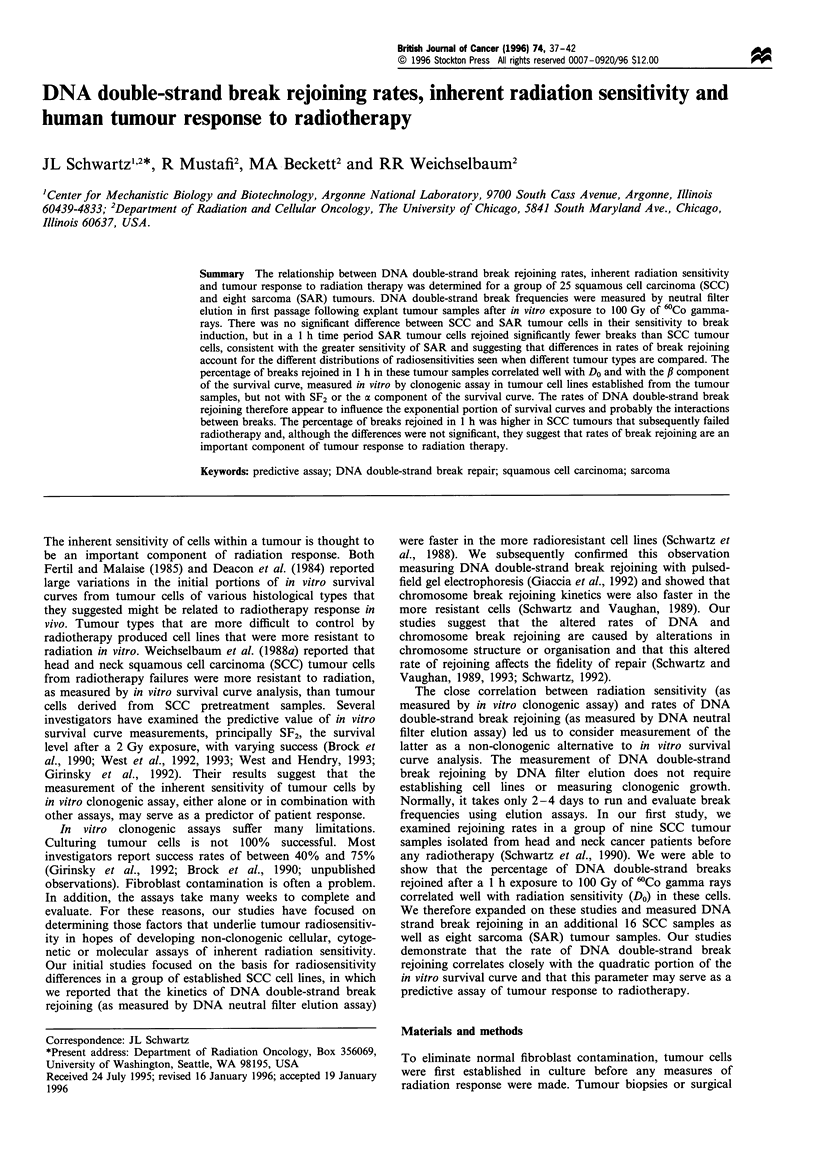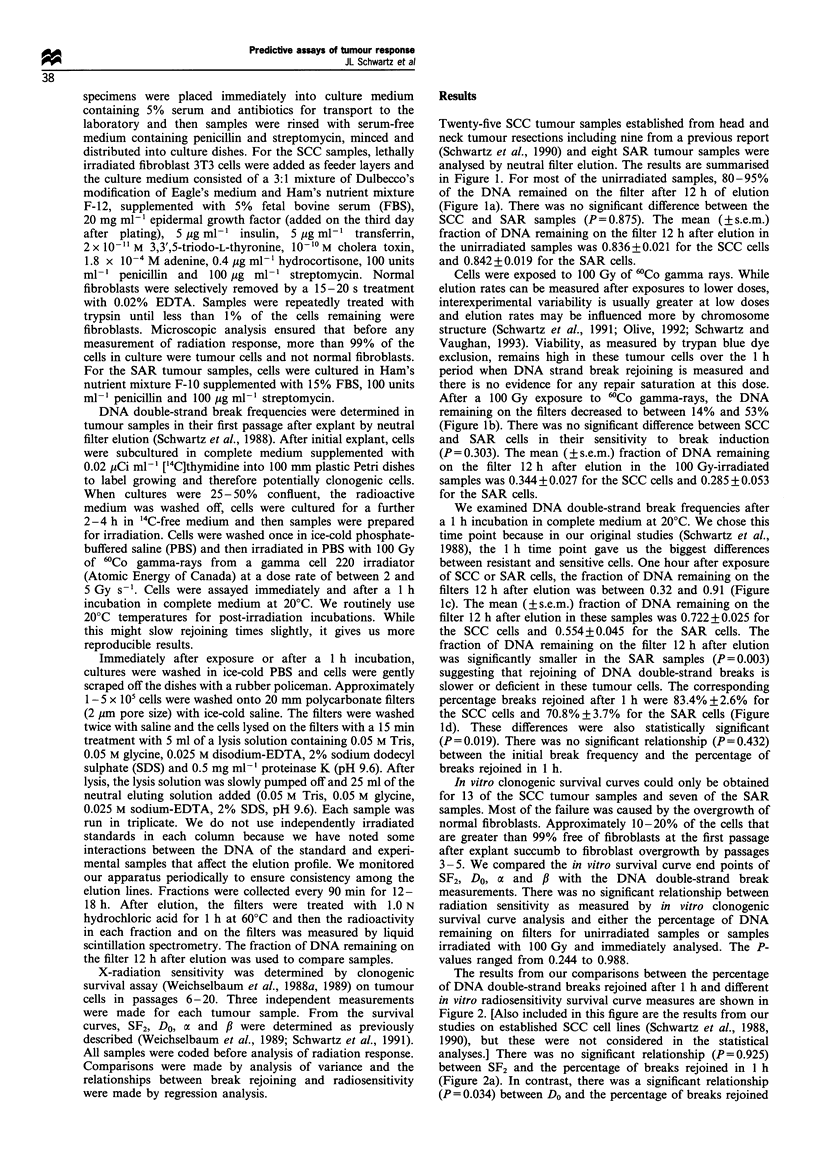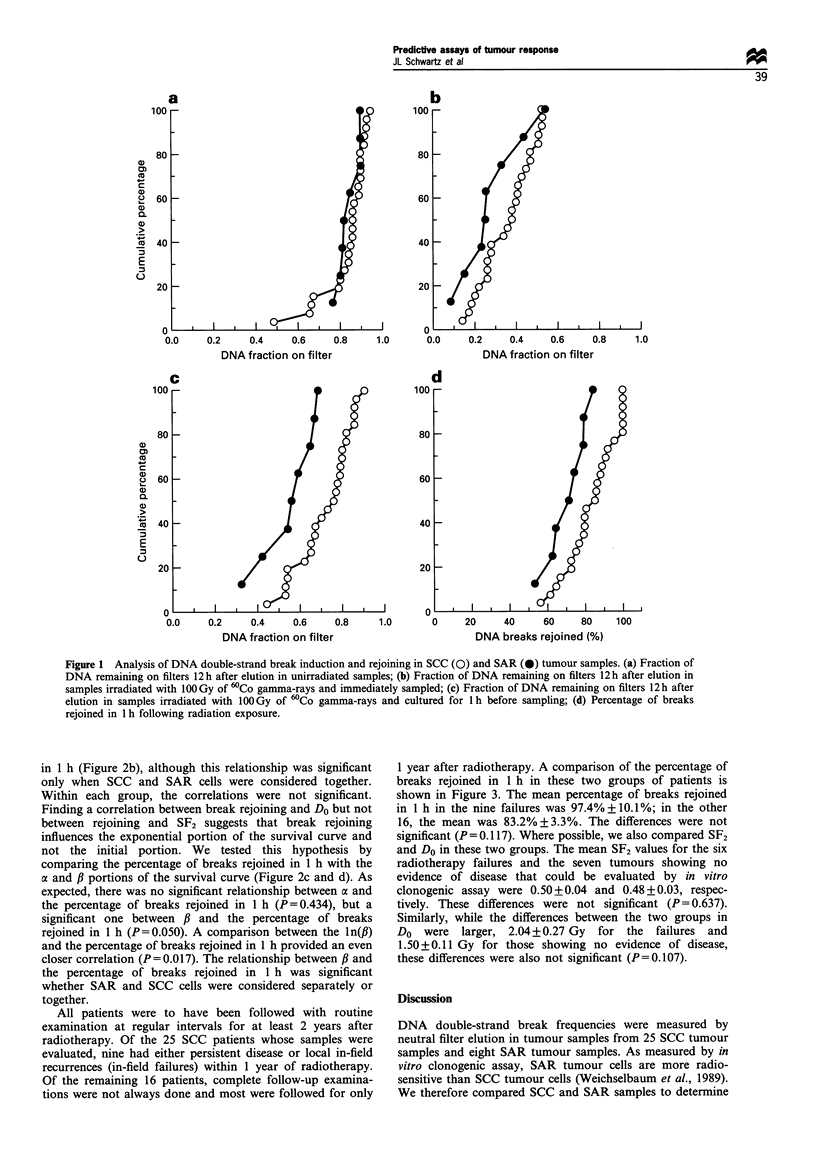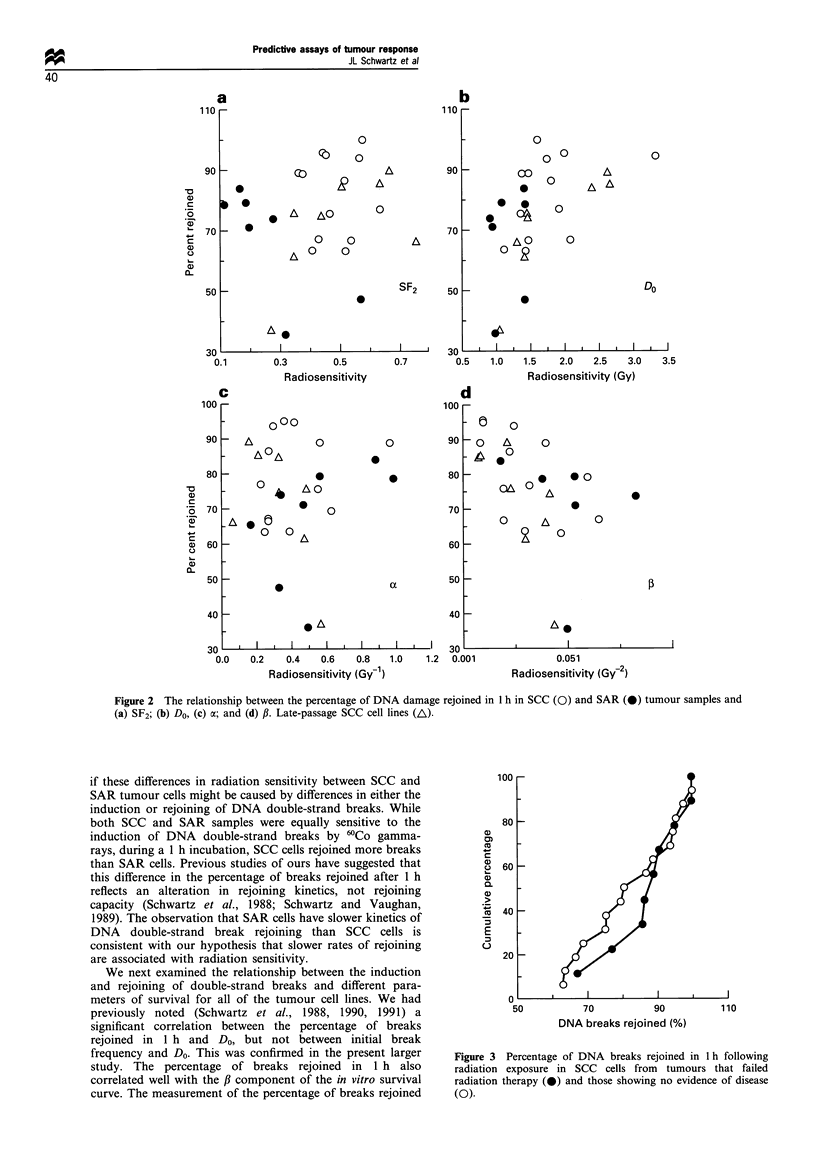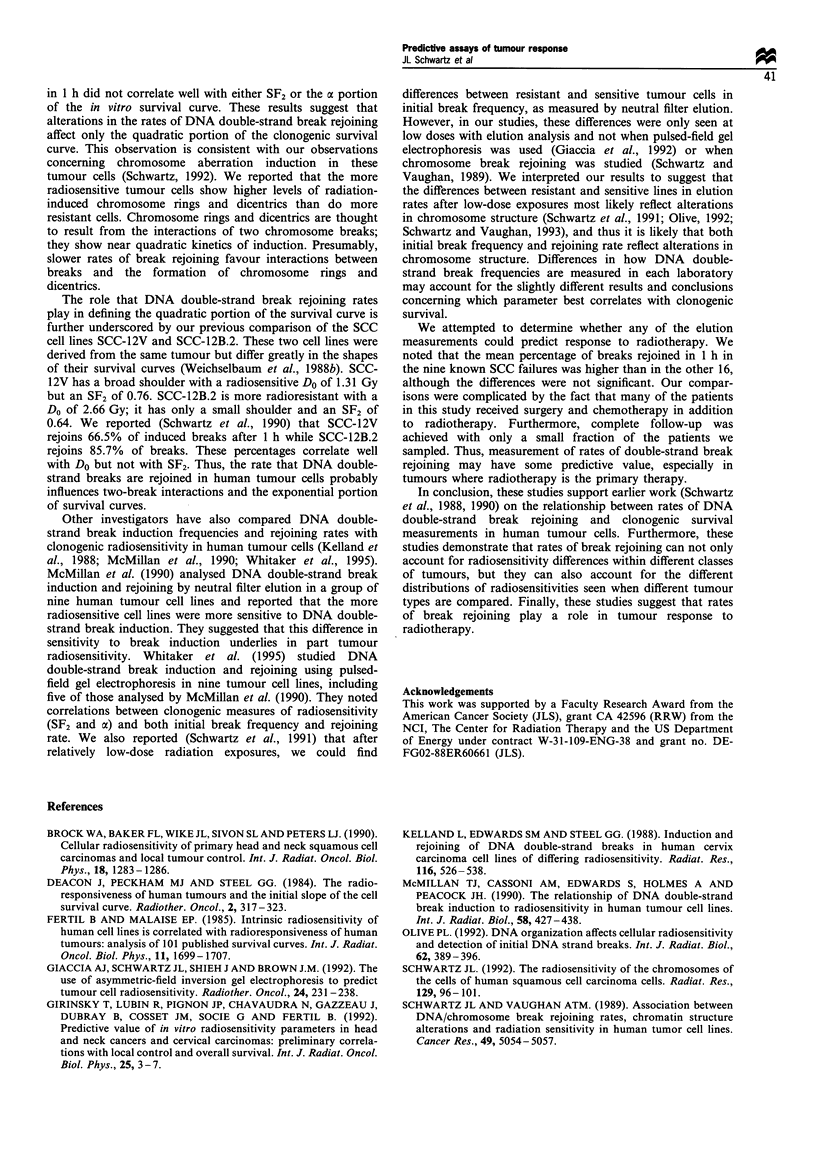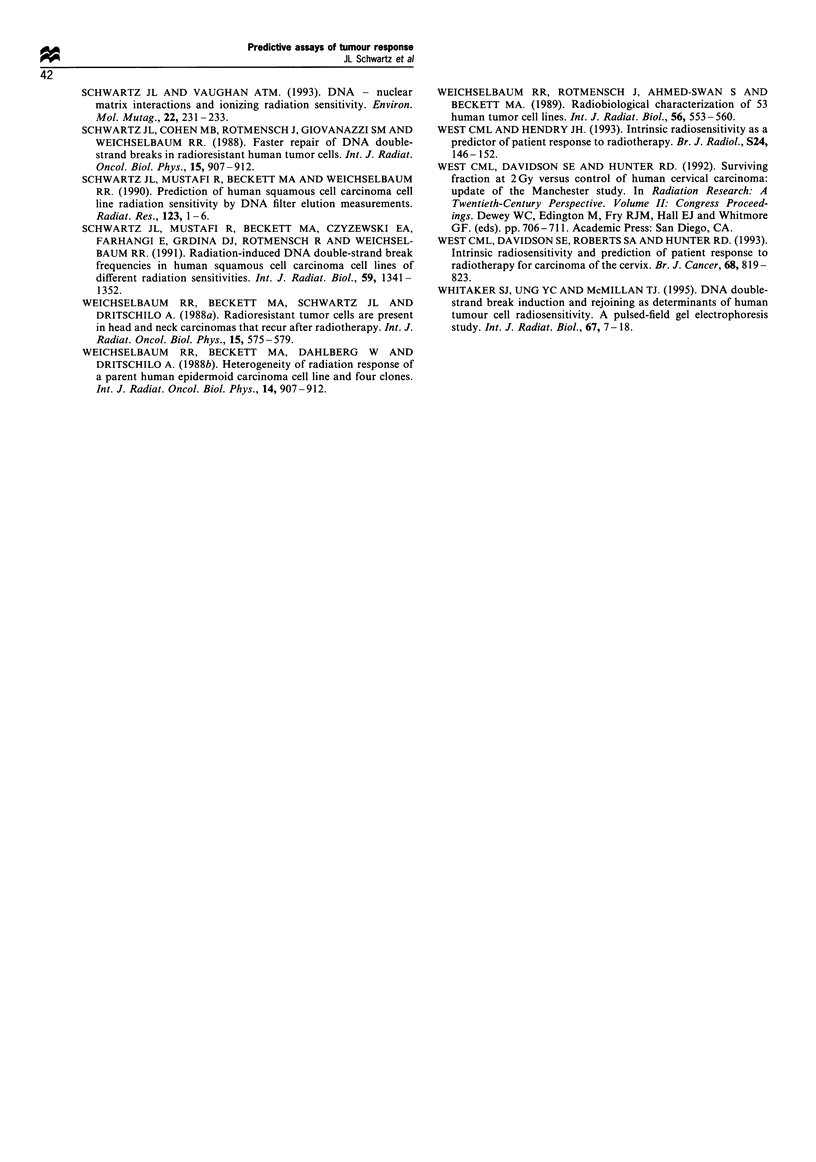# DNA double-strand break rejoining rates, inherent radiation sensitivity and human tumour response to radiotherapy.

**DOI:** 10.1038/bjc.1996.312

**Published:** 1996-07

**Authors:** J. L. Schwartz, R. Mustafi, M. A. Beckett, R. R. Weichselbaum

**Affiliations:** Center for Mechanistic Biology and Biotechnology, Argonne National Laboratory, Illinois 60439-4833, USA.

## Abstract

The relationship between DNA double-strand break rejoining rates, inherent radiation sensitivity and tumour response to radiation therapy was determined for a group of 25 squamous cell carcinoma (SCC) and eight sarcoma (SAR) tumours. DNA double-strand break frequencies were measured by neutral filter elution in first passage following explant tumour samples after in vitro exposure to 100 Gy of 60Co gamma-rays. There was no significant difference between SCC and SAR tumour cells in their sensitivity to break induction, but in a 1 h time period SAR tumour cells rejoined significantly fewer breaks than SCC tumour cells, consistent with the greater sensitivity of SAR and suggesting that differences in rates of break rejoining account for the different distributions of radiosensitivities seen when different tumour types are compared. The percentage of breaks rejoined in 1 h in these tumour samples correlated well with D(o) and with the beta component of the survival curve, measured in vitro by clonogenic assay in tumour cell lines established from the tumour samples, but not with SF2 or the alpha component of the survival curve. The rates of DNA double-strand break rejoining therefore appear to influence the exponential portion of survival curves and probably the interactions between breaks. The percentage of breaks rejoined in 1 h was higher in SCC tumours that subsequently failed radiotherapy and, although the differences were not significant, they suggest that rates of break rejoining are an important component of tumour response to radiation therapy.


British Journal of Cancer (1996) 74, 37-42
© 1996 Stockton Press All rights reserved 0007-0920/96 $12.00

### DNA double-strand break rejoining rates, inherent radiation sensitivity and human tumour response to radiotherapy

JL Schwartz[1,2]*, R Mustafi[2], MA Beckett[2] and RR Weichselbaum[2]

[1]*Center for Mechanistic Biology and Biotechnology, Argonne National Laboratory, 9700 South Cass Avenue, Argonne, Illinois 60439-4833;* [2]*Department of Radiation and Cellular Oncology, The University of Chicago, 5841 South Maryland Ave., Chicago, Illinois 60637, USA.*

**Summary** The relationship between DNA double-strand break rejoining rates, inherent radiation sensitivity and tumour response to radiation therapy was determined for a group of 25 squamous cell carcinoma (SCC) and eight sarcoma (SAR) tumours. DNA double-strand break frequencies were measured by neutral filter elution in first passage following explant tumour samples after *in vitro* exposure to 100 Gy of $^{60}Co$ gamma-rays. There was no significant difference between SCC and SAR tumour cells in their sensitivity to break induction, but in a 1 h time period SAR tumour cells rejoined significantly fewer breaks than SCC tumour cells, consistent with the greater sensitivity of SAR and suggesting that differences in rates of break rejoining account for the different distributions of radiosensitivities seen when different tumour types are compared. The percentage of breaks rejoined in 1 h in these tumour samples correlated well with $D_0$ and with the $\beta$ component of the survival curve, measured *in vitro* by clonogenic assay in tumour cell lines established from the tumour samples, but not with $SF_2$ or the $\alpha$ component of the survival curve. The rates of DNA double-strand break rejoining therefore appear to influence the exponential portion of survival curves and probably the interactions between breaks. The percentage of breaks rejoined in 1 h was higher in SCC tumours that subsequently failed radiotherapy and, although the differences were not significant, they suggest that rates of break rejoining are an important component of tumour response to radiation therapy.

**Keywords:** predictive assay; DNA double-strand break repair; squamous cell carcinoma; sarcoma

The inherent sensitivity of cells within a tumour is thought to be an important component of radiation response. Both Fertil and Malaise (1985) and Deacon *et al.* (1984) reported large variations in the initial portions of *in vitro* survival curves from tumour cells of various histological types that they suggested might be related to radiotherapy response *in vivo*. Tumour types that are more difficult to control by radiotherapy produced cell lines that were more resistant to radiation *in vitro*. Weichselbaum *et al.* (1988*a*) reported that head and neck squamous cell carcinoma (SCC) tumour cells from radiotherapy failures were more resistant to radiation, as measured by *in vitro* survival curve analysis, than tumour cells derived from SCC pretreatment samples. Several investigators have examined the predictive value of *in vitro* survival curve measurements, principally $SF_2$, the survival level after a 2 Gy exposure, with varying success (Brock *et al.*, 1990; West *et al.*, 1992, 1993; West and Hendry, 1993; Girinsky *et al.*, 1992). Their results suggest that the measurement of the inherent sensitivity of tumour cells by *in vitro* clonogenic assay, either alone or in combination with other assays, may serve as a predictor of patient response.

*In vitro* clonogenic assays suffer many limitations. Culturing tumour cells is not 100% successful. Most investigators report success rates of between 40% and 75% (Girinsky *et al.*, 1992; Brock *et al.*, 1990; unpublished observations). Fibroblast contamination is often a problem. In addition, the assays take many weeks to complete and evaluate. For these reasons, our studies have focused on determining those factors that underlie tumour radiosensitivity in hopes of developing non-clonogenic cellular, cytogenetic or molecular assays of inherent radiation sensitivity. Our initial studies focused on the basis for radiosensitivity differences in a group of established SCC cell lines, in which we reported that the kinetics of DNA double-strand break rejoining (as measured by DNA neutral filter elution assay) were faster in the more radioresistant cell lines (Schwartz *et al.*, 1988). We subsequently confirmed this observation measuring DNA double-strand break rejoining with pulsed-field gel electrophoresis (Giaccia *et al.*, 1992) and showed that chromosome break rejoining kinetics were also faster in the more resistant cells (Schwartz and Vaughan, 1989). Our studies suggest that the altered rates of DNA and chromosome break rejoining are caused by alterations in chromosome structure or organisation and that this altered rate of rejoining affects the fidelity of repair (Schwartz and Vaughan, 1989, 1993; Schwartz, 1992).

The close correlation between radiation sensitivity (as measured by *in vitro* clonogenic assay) and rates of DNA double-strand break rejoining (as measured by DNA neutral filter elution assay) led us to consider measurement of the latter as a non-clonogenic alternative to *in vitro* survival curve analysis. The measurement of DNA double-strand break rejoining by DNA filter elution does not require establishing cell lines or measuring clonogenic growth. Normally, it takes only 2-4 days to run and evaluate break frequencies using elution assays. In our first study, we examined rejoining rates in a group of nine SCC tumour samples isolated from head and neck cancer patients before any radiotherapy (Schwartz *et al.*, 1990). We were able to show that the percentage of DNA double-strand breaks rejoined after a 1 h exposure to 100 Gy of $^{60}Co$ gamma rays correlated well with radiation sensitivity ($D_0$) in these cells. We therefore expanded on these studies and measured DNA strand break rejoining in an additional 16 SCC samples as well as eight sarcoma (SAR) tumour samples. Our studies demonstrate that the rate of DNA double-strand break rejoining correlates closely with the quadratic portion of the *in vitro* survival curve and that this parameter may serve as a predictive assay of tumour response to radiotherapy.

### Materials and methods

To eliminate normal fibroblast contamination, tumour cells were first established in culture before any measures of radiation response were made. Tumour biopsies or surgical

Correspondence: JL Schwartz
*Present address: Department of Radiation Oncology, Box 356069, University of Washington, Seattle, WA 98195, USA
Received 24 July 1995; revised 16 January 1996; accepted 19 January 1996

specimens were placed immediately into culture medium containing 5% serum and antibiotics for transport to the laboratory and then samples were rinsed with serum-free medium containing penicillin and streptomycin, minced and distributed into culture dishes. For the SCC samples, lethally irradiated fibroblast 3T3 cells were added as feeder layers and the culture medium consisted of a 3:1 mixture of Dulbecco's modification of Eagle's medium and Ham's nutrient mixture F-12, supplemented with 5% fetal bovine serum (FBS), 20 mg $ml^{-1}$ epidermal growth factor (added on the third day after plating), 5 $\mu g\ ml^{-1}$ insulin, 5 $\mu g\ ml^{-1}$ transferrin, $2 \times 10^{-11}$ M 3,3',5-triodo-L-thyronine, $10^{-10}$ M cholera toxin, $1.8 \times 10^{-4}$ M adenine, 0.4 $\mu g\ ml^{-1}$ hydrocortisone, 100 units $ml^{-1}$ penicillin and 100 $\mu g\ ml^{-1}$ streptomycin. Normal fibroblasts were selectively removed by a 15–20 s treatment with 0.02% EDTA. Samples were repeatedly treated with trypsin until less than 1% of the cells remaining were fibroblasts. Microscopic analysis ensured that before any measurement of radiation response, more than 99% of the cells in culture were tumour cells and not normal fibroblasts. For the SAR tumour samples, cells were cultured in Ham's nutrient mixture F-10 supplemented with 15% FBS, 100 units $ml^{-1}$ penicillin and 100 $\mu g\ ml^{-1}$ streptomycin.

DNA double-strand break frequencies were determined in tumour samples in their first passage after explant by neutral filter elution (Schwartz *et al.*, 1988). After initial explant, cells were subcultured in complete medium supplemented with 0.02 $\mu Ci\ ml^{-1}$ [$^{14}$C]thymidine into 100 mm plastic Petri dishes to label growing and therefore potentially clonogenic cells. When cultures were 25–50% confluent, the radioactive medium was washed off, cells were cultured for a further 2–4 h in $^{14}$C-free medium and then samples were prepared for irradiation. Cells were washed once in ice-cold phosphate-buffered saline (PBS) and then irradiated in PBS with 100 Gy of $^{60}$Co gamma-rays from a gamma cell 220 irradiator (Atomic Energy of Canada) at a dose rate of between 2 and 5 Gy $s^{-1}$. Cells were assayed immediately and after a 1 h incubation in complete medium at 20°C. We routinely use 20°C temperatures for post-irradiation incubations. While this might slow rejoining times slightly, it gives us more reproducible results.

Immediately after exposure or after a 1 h incubation, cultures were washed in ice-cold PBS and cells were gently scraped off the dishes with a rubber policeman. Approximately $1-5 \times 10^5$ cells were washed onto 20 mm polycarbonate filters (2 $\mu$m pore size) with ice-cold saline. The filters were washed twice with saline and the cells lysed on the filters with a 15 min treatment with 5 ml of a lysis solution containing 0.05 M Tris, 0.05 M glycine, 0.025 M disodium-EDTA, 2% sodium dodecyl sulphate (SDS) and 0.5 mg $ml^{-1}$ proteinase K (pH 9.6). After lysis, the lysis solution was slowly pumped off and 25 ml of the neutral eluting solution added (0.05 M Tris, 0.05 M glycine, 0.025 M sodium-EDTA, 2% SDS, pH 9.6). Each sample was run in triplicate. We do not use independently irradiated standards in each column because we have noted some interactions between the DNA of the standard and experimental samples that affect the elution profile. We monitored our apparatus periodically to ensure consistency among the elution lines. Fractions were collected every 90 min for 12–18 h. After elution, the filters were treated with 1.0 N hydrochloric acid for 1 h at 60°C and then the radioactivity in each fraction and on the filters was measured by liquid scintillation spectrometry. The fraction of DNA remaining on the filter 12 h after elution was used to compare samples.

X-radiation sensitivity was determined by clonogenic survival assay (Weichselbaum *et al.*, 1988*a*, 1989) on tumour cells in passages 6–20. Three independent measurements were made for each tumour sample. From the survival curves, $SF_2$, $D_0$, $\alpha$ and $\beta$ were determined as previously described (Weichselbaum *et al.*, 1989; Schwartz *et al.*, 1991). All samples were coded before analysis of radiation response. Comparisons were made by analysis of variance and the relationships between break rejoining and radiosensitivity were made by regression analysis.

### Results

Twenty-five SCC tumour samples established from head and neck tumour resections including nine from a previous report (Schwartz *et al.*, 1990) and eight SAR tumour samples were analysed by neutral filter elution. The results are summarised in Figure 1. For most of the unirradiated samples, 80–95% of the DNA remained on the filter after 12 h of elution (Figure 1a). There was no significant difference between the SCC and SAR samples ($P = 0.875$). The mean ($\pm$s.e.m.) fraction of DNA remaining on the filter 12 h after elution in the unirradiated samples was $0.836 \pm 0.021$ for the SCC cells and $0.842 \pm 0.019$ for the SAR cells.

Cells were exposed to 100 Gy of $^{60}$Co gamma rays. While elution rates can be measured after exposures to lower doses, interexperimental variability is usually greater at low doses and elution rates may be influenced more by chromosome structure (Schwartz *et al.*, 1991; Olive, 1992; Schwartz and Vaughan, 1993). Viability, as measured by trypan blue dye exclusion, remains high in these tumour cells over the 1 h period when DNA strand break rejoining is measured and there is no evidence for any repair saturation at this dose. After a 100 Gy exposure to $^{60}$Co gamma-rays, the DNA remaining on the filters decreased to between 14% and 53% (Figure 1b). There was no significant difference between SCC and SAR cells in their sensitivity to break induction ($P = 0.303$). The mean ($\pm$s.e.m.) fraction of DNA remaining on the filter 12 h after elution in the 100 Gy-irradiated samples was $0.344 \pm 0.027$ for the SCC cells and $0.285 \pm 0.053$ for the SAR cells.

We examined DNA double-strand break frequencies after a 1 h incubation in complete medium at 20°C. We chose this time point because in our original studies (Schwartz *et al.*, 1988), the 1 h time point gave us the biggest differences between resistant and sensitive cells. One hour after exposure of SCC or SAR cells, the fraction of DNA remaining on the filters 12 h after elution was between 0.32 and 0.91 (Figure 1c). The mean ($\pm$s.e.m.) fraction of DNA remaining on the filter 12 h after elution in these samples was $0.722 \pm 0.025$ for the SCC cells and $0.554 \pm 0.045$ for the SAR cells. The fraction of DNA remaining on the filter 12 h after elution was significantly smaller in the SAR samples ($P = 0.003$) suggesting that rejoining of DNA double-strand breaks is slower or deficient in these tumour cells. The corresponding percentage breaks rejoined after 1 h were $83.4\% \pm 2.6\%$ for the SCC cells and $70.8\% \pm 3.7\%$ for the SAR cells (Figure 1d). These differences were also statistically significant ($P = 0.019$). There was no significant relationship ($P = 0.432$) between the initial break frequency and the percentage of breaks rejoined in 1 h.

*In vitro* clonogenic survival curves could only be obtained for 13 of the SCC tumour samples and seven of the SAR samples. Most of the failure was caused by the overgrowth of normal fibroblasts. Approximately 10–20% of the cells that are greater than 99% free of fibroblasts at the first passage after explant succumb to fibroblast overgrowth by passages 3–5. We compared the *in vitro* survival curve end points of $SF_2$, $D_0$, $\alpha$ and $\beta$ with the DNA double-strand break measurements. There was no significant relationship between radiation sensitivity as measured by *in vitro* clonogenic survival curve analysis and either the percentage of DNA remaining on filters for unirradiated samples or samples irradiated with 100 Gy and immediately analysed. The *P*-values ranged from 0.244 to 0.988.

The results from our comparisons between the percentage of DNA double-strand breaks rejoined after 1 h and different *in vitro* radiosensitivity survival curve measures are shown in Figure 2. [Also included in this figure are the results from our studies on established SCC cell lines (Schwartz *et al.*, 1988, 1990), but these were not considered in the statistical analyses.] There was no significant relationship ($P = 0.925$) between $SF_2$ and the percentage of breaks rejoined in 1 h (Figure 2a). In contrast, there was a significant relationship ($P = 0.034$) between $D_0$ and the percentage of breaks rejoined

**Figure 1** Analysis of DNA double-strand break induction and rejoining in SCC (○) and SAR (●) tumour samples. (**a**) Fraction of DNA remaining on filters 12 h after elution in unirradiated samples; (**b**) Fraction of DNA remaining on filters 12 h after elution in samples irradiated with 100 Gy of $^{60}Co$ gamma-rays and immediately sampled; (**c**) Fraction of DNA remaining on filters 12 h after elution in samples irradiated with 100 Gy of $^{60}Co$ gamma-rays and cultured for 1 h before sampling; (**d**) Percentage of breaks rejoined in 1 h following radiation exposure.

in 1 h (Figure 2b), although this relationship was significant only when SCC and SAR cells were considered together. Within each group, the correlations were not significant. Finding a correlation between break rejoining and $D_0$ but not between rejoining and $SF_2$ suggests that break rejoining influences the exponential portion of the survival curve and not the initial portion. We tested this hypothesis by comparing the percentage of breaks rejoined in 1 h with the $\alpha$ and $\beta$ portions of the survival curve (Figure 2c and d). As expected, there was no significant relationship between $\alpha$ and the percentage of breaks rejoined in 1 h ($P=0.434$), but a significant one between $\beta$ and the percentage of breaks rejoined in 1 h ($P=0.050$). A comparison between the $\ln(\beta)$ and the percentage of breaks rejoined in 1 h provided an even closer correlation ($P=0.017$). The relationship between $\beta$ and the percentage of breaks rejoined in 1 h was significant whether SAR and SCC cells were considered separately or together.

All patients were to have been followed with routine examination at regular intervals for at least 2 years after radiotherapy. Of the 25 SCC patients whose samples were evaluated, nine had either persistent disease or local in-field recurrences (in-field failures) within 1 year of radiotherapy. Of the remaining 16 patients, complete follow-up examinations were not always done and most were followed for only 1 year after radiotherapy. A comparison of the percentage of breaks rejoined in 1 h in these two groups of patients is shown in Figure 3. The mean percentage of breaks rejoined in 1 h in the nine failures was $97.4\% \pm 10.1\%$; in the other 16, the mean was $83.2\% \pm 3.3\%$. The differences were not significant ($P=0.117$). Where possible, we also compared $SF_2$ and $D_0$ in these two groups. The mean $SF_2$ values for the six radiotherapy failures and the seven tumours showing no evidence of disease that could be evaluated by *in vitro* clonogenic assay were $0.50 \pm 0.04$ and $0.48 \pm 0.03$, respectively. These differences were not significant ($P=0.637$). Similarly, while the differences between the two groups in $D_0$ were larger, $2.04 \pm 0.27$ Gy for the failures and $1.50 \pm 0.11$ Gy for those showing no evidence of disease, these differences were also not significant ($P=0.107$).

### Discussion

DNA double-strand break frequencies were measured by neutral filter elution in tumour samples from 25 SCC tumour samples and eight SAR tumour samples. As measured by *in vitro* clonogenic assay, SAR tumour cells are more radiosensitive than SCC tumour cells (Weichselbaum *et al*., 1989). We therefore compared SCC and SAR samples to determine

**Figure 2** The relationship between the percentage of DNA damage rejoined in 1 h in SCC (○) and SAR (●) tumour samples and (**a**) $SF_2$; (**b**) $D_0$, (**c**) $\alpha$; and (**d**) $\beta$. Late-passage SCC cell lines (△).

if these differences in radiation sensitivity between SCC and SAR tumour cells might be caused by differences in either the induction or rejoining of DNA double-strand breaks. While both SCC and SAR samples were equally sensitive to the induction of DNA double-strand breaks by $^{60}Co$ gamma-rays, during a 1 h incubation, SCC cells rejoined more breaks than SAR cells. Previous studies of ours have suggested that this difference in the percentage of breaks rejoined after 1 h reflects an alteration in rejoining kinetics, not rejoining capacity (Schwartz *et al.*, 1988; Schwartz and Vaughan, 1989). The observation that SAR cells have slower kinetics of DNA double-strand break rejoining than SCC cells is consistent with our hypothesis that slower rates of rejoining are associated with radiation sensitivity.

We next examined the relationship between the induction and rejoining of double-strand breaks and different parameters of survival for all of the tumour cell lines. We had previously noted (Schwartz *et al.*, 1988, 1990, 1991) a significant correlation between the percentage of breaks rejoined in 1 h and $D_0$, but not between initial break frequency and $D_0$. This was confirmed in the present larger study. The percentage of breaks rejoined in 1 h also correlated well with the $\beta$ component of the *in vitro* survival curve. The measurement of the percentage of breaks rejoined

**Figure 3** Percentage of DNA breaks rejoined in 1 h following radiation exposure in SCC cells from tumours that failed radiation therapy (●) and those showing no evidence of disease (○).

in 1 h did not correlate well with either $SF_2$ or the $\alpha$ portion of the *in vitro* survival curve. These results suggest that alterations in the rates of DNA double-strand break rejoining affect only the quadratic portion of the clonogenic survival curve. This observation is consistent with our observations concerning chromosome aberration induction in these tumour cells (Schwartz, 1992). We reported that the more radiosensitive tumour cells show higher levels of radiation-induced chromosome rings and dicentrics than do more resistant cells. Chromosome rings and dicentrics are thought to result from the interactions of two chromosome breaks; they show near quadratic kinetics of induction. Presumably, slower rates of break rejoining favour interactions between breaks and the formation of chromosome rings and dicentrics.

The role that DNA double-strand break rejoining rates play in defining the quadratic portion of the survival curve is further underscored by our previous comparison of the SCC cell lines SCC-12V and SCC-12B.2. These two cell lines were derived from the same tumour but differ greatly in the shapes of their survival curves (Weichselbaum *et al.*, 1988*b*). SCC-12V has a broad shoulder with a radiosensitive $D_0$ of 1.31 Gy but an $SF_2$ of 0.76. SCC-12B.2 is more radioresistant with a $D_0$ of 2.66 Gy; it has only a small shoulder and an $SF_2$ of 0.64. We reported (Schwartz *et al.*, 1990) that SCC-12V rejoins 66.5% of induced breaks after 1 h while SCC-12B.2 rejoins 85.7% of breaks. These percentages correlate well with $D_0$ but not with $SF_2$. Thus, the rate that DNA double-strand breaks are rejoined in human tumour cells probably influences two-break interactions and the exponential portion of survival curves.

Other investigators have also compared DNA double-strand break induction frequencies and rejoining rates with clonogenic radiosensitivity in human tumour cells (Kelland *et al.*, 1988; McMillan *et al.*, 1990; Whitaker *et al.*, 1995). McMillan *et al.* (1990) analysed DNA double-strand break induction and rejoining by neutral filter elution in a group of nine human tumour cell lines and reported that the more radiosensitive cell lines were more sensitive to DNA double-strand break induction. They suggested that this difference in sensitivity to break induction underlies in part tumour radiosensitivity. Whitaker *et al.* (1995) studied DNA double-strand break induction and rejoining using pulsed-field gel electrophoresis in nine tumour cell lines, including five of those analysed by McMillan *et al.* (1990). They noted correlations between clonogenic measures of radiosensitivity ($SF_2$ and $\alpha$) and both initial break frequency and rejoining rate. We also reported (Schwartz *et al.*, 1991) that after relatively low-dose radiation exposures, we could find differences between resistant and sensitive tumour cells in initial break frequency, as measured by neutral filter elution. However, in our studies, these differences were only seen at low doses with elution analysis and not when pulsed-field gel electrophoresis was used (Giaccia *et al.*, 1992) or when chromosome break rejoining was studied (Schwartz and Vaughan, 1989). We interpreted our results to suggest that the differences between resistant and sensitive lines in elution rates after low-dose exposures most likely reflect alterations in chromosome structure (Schwartz *et al.*, 1991; Olive, 1992; Schwartz and Vaughan, 1993), and thus it is likely that both initial break frequency and rejoining rate reflect alterations in chromosome structure. Differences in how DNA double-strand break frequencies are measured in each laboratory may account for the slightly different results and conclusions concerning which parameter best correlates with clonogenic survival.

We attempted to determine whether any of the elution measurements could predict response to radiotherapy. We noted that the mean percentage of breaks rejoined in 1 h in the nine known SCC failures was higher than in the other 16, although the differences were not significant. Our comparisons were complicated by the fact that many of the patients in this study received surgery and chemotherapy in addition to radiotherapy. Furthermore, complete follow-up was achieved with only a small fraction of the patients we sampled. Thus, measurement of rates of double-strand break rejoining may have some predictive value, especially in tumours where radiotherapy is the primary therapy.

In conclusion, these studies support earlier work (Schwartz *et al.*, 1988, 1990) on the relationship between rates of DNA double-strand break rejoining and clonogenic survival measurements in human tumour cells. Furthermore, these studies demonstrate that rates of break rejoining can not only account for radiosensitivity differences within different classes of tumours, but they can also account for the different distributions of radiosensitivities seen when different tumour types are compared. Finally, these studies suggest that rates of break rejoining play a role in tumour response to radiotherapy.

**Acknowledgements**
This work was supported by a Faculty Research Award from the American Cancer Society (JLS), grant CA 42596 (RRW) from the NCI, The Center for Radiation Therapy and the US Department of Energy under contract W-31-109-ENG-38 and grant no. DE-FG02-88ER60661 (JLS).

### References

BROCK WA, BAKER FL, WIKE JL, SIVON SL AND PETERS LJ. (1990). Cellular radiosensitivity of primary head and neck squamous cell carcinomas and local tumour control. *Int. J. Radiat. Oncol. Biol. Phys.*, **18**, 1283–1286.

DEACON J, PECKHAM MJ AND STEEL GG. (1984). The radioresponsiveness of human tumours and the initial slope of the cell survival curve. *Radiother. Oncol.*, **2**, 317–323.

FERTIL B AND MALAISE EP. (1985). Intrinsic radiosensitivity of human cell lines is correlated with radioresponsiveness of human tumours: analysis of 101 published survival curves. *Int. J. Radiat. Oncol. Biol. Phys.*, **11**, 1699–1707.

GIACCIA AJ, SCHWARTZ JL, SHIEH J AND BROWN J.M. (1992). The use of asymmetric-field inversion gel electrophoresis to predict tumour cell radiosensitivity. *Radiother. Oncol.*, **24**, 231–238.

GIRINSKY T, LUBIN R, PIGNON JP, CHAVAUDRA N, GAZZEAU J, DUBRAY B, COSSET JM, SOCIE G AND FERTIL B. (1992). Predictive value of *in vitro* radiosensitivity parameters in head and neck cancers and cervical carcinomas: preliminary correlations with local control and overall survival. *Int. J. Radiat. Oncol. Biol. Phys.*, **25**, 3–7.

KELLAND L, EDWARDS SM AND STEEL GG. (1988). Induction and rejoining of DNA double-strand breaks in human cervix carcinoma cell lines of differing radiosensitivity. *Radiat. Res.*, **116**, 526–538.

McMILLAN TJ, CASSONI AM, EDWARDS S, HOLMES A AND PEACOCK JH. (1990). The relationship of DNA double-strand break induction to radiosensitivity in human tumour cell lines. *Int. J. Radiat. Biol.*, **58**, 427–438.

OLIVE PL. (1992). DNA organization affects cellular radiosensitivity and detection of initial DNA strand breaks. *Int. J. Radiat. Biol.*, **62**, 389–396.

SCHWARTZ JL. (1992). The radiosensitivity of the chromosomes of the cells of human squamous cell carcinoma cells. *Radiat. Res.*, **129**, 96–101.

SCHWARTZ JL AND VAUGHAN ATM. (1989). Association between DNA/chromosome break rejoining rates, chromatin structure alterations and radiation sensitivity in human tumor cell lines. *Cancer Res.*, **49**, 5054–5057.

SCHWARTZ JL AND VAUGHAN ATM. (1993). DNA – nuclear matrix interactions and ionizing radiation sensitivity. *Environ. Mol. Mutag.*, **22**, 231–233.
SCHWARTZ JL, COHEN MB, ROTMENSCH J, GIOVANAZZI SM AND WEICHSELBAUM RR. (1988). Faster repair of DNA double-strand breaks in radioresistant human tumor cells. *Int. J. Radiat. Oncol. Biol. Phys.*, **15**, 907–912.
SCHWARTZ JL, MUSTAFI R, BECKETT MA AND WEICHSELBAUM RR. (1990). Prediction of human squamous cell carcinoma cell line radiation sensitivity by DNA filter elution measurements. *Radiat. Res.*, **123**, 1–6.
SCHWARTZ JL, MUSTAFI R, BECKETT MA, CZYZEWSKI EA, FARHANGI E, GRDINA DJ, ROTMENSCH R AND WEICHSELBAUM RR. (1991). Radiation-induced DNA double-strand break frequencies in human squamous cell carcinoma cell lines of different radiation sensitivities. *Int. J. Radiat. Biol.*, **59**, 1341–1352.
WEICHSELBAUM RR, BECKETT MA, SCHWARTZ JL AND DRITSCHILO A. (1988*a*). Radioresistant tumor cells are present in head and neck carcinomas that recur after radiotherapy. *Int. J. Radiat. Oncol. Biol. Phys.*, **15**, 575–579.
WEICHSELBAUM RR, BECKETT MA, DAHLBERG W AND DRITSCHILO A. (1988*b*). Heterogeneity of radiation response of a parent human epidermoid carcinoma cell line and four clones. *Int. J. Radiat. Oncol. Biol. Phys.*, **14**, 907–912.
WEICHSELBAUM RR, ROTMENSCH J, AHMED-SWAN S AND BECKETT MA. (1989). Radiobiological characterization of 53 human tumor cell lines. *Int. J. Radiat. Biol.*, **56**, 553–560.
WEST CML AND HENDRY JH. (1993). Intrinsic radiosensitivity as a predictor of patient response to radiotherapy. *Br. J. Radiol.*, **S24**, 146–152.
WEST CML, DAVIDSON SE AND HUNTER RD. (1992). Surviving fraction at 2 Gy versus control of human cervical carcinoma: update of the Manchester study. In *Radiation Research: A Twentieth-Century Perspective. Volume II: Congress Proceedings*. Dewey WC, Edington M, Fry RJM, Hall EJ and Whitmore GF. (eds). pp. 706–711. Academic Press: San Diego, CA.
WEST CML, DAVIDSON SE, ROBERTS SA AND HUNTER RD. (1993). Intrinsic radiosensitivity and prediction of patient response to radiotherapy for carcinoma of the cervix. *Br. J. Cancer*, **68**, 819–823.
WHITAKER SJ, UNG YC AND MCMILLAN TJ. (1995). DNA double-strand break induction and rejoining as determinants of human tumour cell radiosensitivity. A pulsed-field gel electrophoresis study. *Int. J. Radiat. Biol.*, **67**, 7–18.